# Hypothermia Induced by Adenosine 5′-Monophosphate Attenuates Acute Lung Injury Induced by LPS in Rats

**DOI:** 10.1155/2012/459617

**Published:** 2012-09-13

**Authors:** Zhimin Miao, Shulai Lu, Na Du, Weiting Guo, Jidong Zhang, Yu Song, Xuefeng Wang, Wei Ren, Yunlong Wang, Tao Jiang

**Affiliations:** ^1^Gout Laboratory, The Affiliated Hospital of Medical College Qingdao University, Shandong Provincial Key Laboratory of Metabolic Diseases, 16 Jiangsu Road, Qingdao 266003, China; ^2^Stomatological Department, Qingdao Municipal Hospital, Qingdao 266011, China; ^3^Key Laboratory of Marine Drugs, School of Pharmacy, Ocean University of China, Ministry of Education of China, Qingdao 266003, China; ^4^Department of Cardiology, The Affiliated Hospital of Medical College Qingdao University, Qingdao 266011, China

## Abstract

We have built a rat's model to investigate whether the hypothermia induced by adenosine 5′-monophosphate (5′-AMP) (AIH) could attenuate acute lung injury induced by LPS in rats. We detected the inflammatory cytokine levels in the plasma and bronchoalveolar lavage fluid samples, and we analyzed the pathological changes in the lungs. We have found that AIH can effectively inhibit acute inflammatory reactions and protect the lung from acute injury induced by LPS in rats.

## 1. Introduction

Lipopolysaccharides (LPSs) are the main components of the outer membrane of gram-negative bacteria and act as basic mediators that host the inflammatory sequelae after a gram-negative bacterial infection. Consisting of four different parts, including lipid A, the inner core, the outer core, and the O antigen [1], LPS is nontoxic when it functions as a component of the bacterial cell wall. However, it becomes the opposite when it is released from the cell wall as the cells multiply or die due to its toxic component, lipid A [1]. After the host's immune system is exposed to lipid A, an inflammatory response is evoked. Upon binding with LPS-binding protein (LBP) in the plasma, the LPS-LBP complex integrates into the cell surface and activates the CD14 receptor, where LPS is then delivered to the transmembrane signaling receptor toll-like receptor 4 [1].

Following the infection, endotoxemia or endotoxic shock is induced, which are characterized by a cascade of cytokines that are expressed and released, such as tumor necrosis factor-*α* (TNF-*α*), interleukin-1*β* (IL-1*β*), and IL-6, followed by an anti-inflammatory response with the production of anti-inflammatory cytokines, such as IL-10 [2, 3]. Both endotoxins and cytokines contribute to the pathophysiology of endotoxic shock and the development of organ injury in endotoxemia, such as acute lung injury [4, 5]. It has been shown that hypothermia can reduce the mortality in the murine endotoxic shock model [6]. One of the possible mechanisms for this reduction may be decreased oxygen demand, which can contribute to organ preservation. Another possibility is a decrease in the levels of proinflammatory cytokines and an increase in the levels of anti-inflammatory cytokines in the plasma [4, 5, 7].

5′-AMP, a biomolecule that was recently discovered, allows nonhibernating mammals to enter a rapid and safe severe hypothermia. It is the first endogenous biomolecule found to have this effect [8]. In mice, 5′-AMP can induce torpor when their core body temperature falls to 31.8°C or below [9–11]. The torpor duration also appears to be dependent on the 5′-AMP dosage [8, 10, 11]. The mechanism of AIH is still unclear. Studies have reported that hypometabolism and a decreased oxygen affinity by the erythrocytes caused by the excessive 5′-AMP influx may represent the mechanism of AIH [9].

 Recently, studies have shown that hypothermia induced by 5′-AMP has an anti-inflammatory effect [9]; however, the anti-inflammatory mechanism of 5′-AMP-induced hypothermia remains unclear. A possible mechanism may be that the hypothermia makes it difficult for NF*κ*B to penetrate the nuclear membrane, thereby reducing its activity. The subsequent inhibition of NF*κ*B's activity then leads to down-regulation of the expression of inflammatory cytokines. Therefore, it is conceivable that the reduction in NF*κ*B activity caused by membrane changes inhibits the inflammatory response of acute lung injury [12].

In this study, we sought to determine whether AMP-induced hypothermia could protect rats from inflammation and acute lung injury induced by LPS.

## 2. Materials and Methods

### 2.1. Materials

Wister rats (158–162 g) were purchased from Shandong Lukang Pharmaceutical Group Co., Ltd. All animal experiments were permitted by the Animal Care Committee of the Affiliated Hospital of Qingdao University Medical College. The adenosine 5′-monophosphate and lipopolysaccharides were purchased from Sigma. IL-1*β*, IL-6, IL-10, and TNF-*α* ELISA kits were purchased from R&D.

### 2.2. Experimental Group

The rats were divided into four groups: (1) control group, in which the animals were not treated; (2) LPS group, in which the rats were treated with LPS only; (3) 5′-AMP pretreatment group (pre-AMP), in which the rats were first treated with 5′-AMP followed by treatment with LPS; (4) 5′-AMP posttreatment group (post-AMP), in which the rats first received LPS followed by treatment with 5′-AMP. Each experimental group was divided into three time points (3 h, 6 h, and 12 h after the treatment with LPS) with 6 rats in each group (*n* = 6).

### 2.3. Construction of a Rat Model of Hypothermia

Both solutions were prepared under pyrogen-free conditions. Endotoxin-free phosphate buffered saline (PBS) was used to dissolve the drugs. The 5′-AMP was suspended in PBS, and the pH was adjusted to 7.4. The freshly prepared 5′-AMP solution was injected i.p. at dosages of 0.125, 0.25, and 0.5 g/kg of body weight to establish a hypothermic model with an ambient temperature (AT) of 16°C.

### 2.4. The TNF-*α*, IL-1*β*, IL-6, and IL-10 Levels in the Plasma and BALF

In the pre-AMP group, freshly prepared 5′-AMP solution was injected (i.p.) at a dosage of 0.5 g/kg of body weight to establish a hypothermic model with an ambient temperature of 16°C. One hour after the 5′-AMP injection, LPS was injected (i.p.) at a dosage of 5 mg/kg of body weight. In the post-AMP group, LPS was injected (i.p.) first, at a dosage of 5 mg/kg of body weight, and when the anal temperature increased by 0.5°C, freshly prepared 5′-AMP solution was injected (i.p.) at a dosage of 0.5 g/kg of body weight to establish a hypothermic model with an ambient temperature of 16°C. The blood was obtained from the ocular venous plexus of the rats in all groups at 3 h, 6 h, and 12 h post-LPS injection, and the plasma was isolated by centrifugation at 12,000 g for 10 min and stored at −80°C until the analyses were performed. The plasma levels of IL-1*β*, IL-6, TNF-*α*, and IL-10 were measured using ELISA kits, according to the manufacturer's instructions. 

Following euthanasia, each rat's right lung was treated with surgical ligation. The left lungs were lavaged three times through a tracheal cannula with 2.5 mL of physiological saline at 3 h, 6 h, and 12 h post-LPS injection. The retrieval volume was maximized by compression of the thorax following the final lavage, and the bronchoalveolar lavage fluid (BALF) was recycled to a total volume of 6.5 mL. The BALF was centrifuged at 3,000 rpm at 4°C for 10 min and stored at −80°C until the analyses were performed. The levels of IL-1*β*, IL-6, TNF-*α*, and IL-10 were measured in the BALF samples using ELISA kits, according to the manufacturer's instructions.

### 2.5. The Hematoxylin-Eosin Staining Analysis of the Lung

Following euthanasia, the lungs were harvested and rinsed free of blood with PBS. The tissues were fixed in 10% neutral formalin and embedded in paraffin. The pulmonary tissue slides were stained with hematoxylin and eosin and were examined using a light microscope (Nikon Ti). Histological judgment was performed in a blind assessment by pathologists. 

## 3. Statistical Analysis

The statistical analyses were performed using the SPSS 10.0 program. The data were presented as means ± standard deviations (SDs) and analyzed using one-way ANOVA and LSD, post hoc. The overall statistical significance was determined as *P *values < 0.05.

## 4. Results

### 4.1. Rat Hypothermia Model Was Induced by 5′-AMP

**Figure 1:**
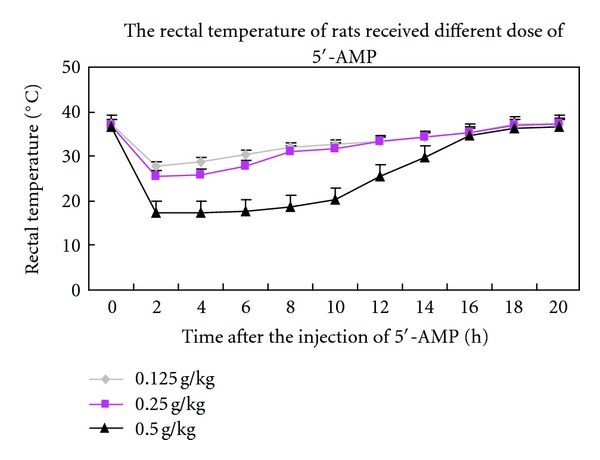
Construction of a rat model of hypothermia. The rectal temperature of the rats receiving different doses of 5′-AMP.

To determine whether 5′-AMP could induce hypothermia in rats and its appropriate dose, rats were injected with 5′-AMP at doses of 0.125, 0.25, or 0.5 g/kg of body weight (i.p.). The rectal temperature of rats that received the 0.125 and 0.25 g/kg dosages decreased to 27.63 ± 1.13°C and 25.55 ± 0.92°C, respectively and was maintained for only a few hours (Figure 1). We found that the rats that received the 0.5 g/kg dose entered a state of torpor (the rectal temperature was lowered to 16.47 ± 1.58°C) approximately 90 min after the injection and maintained a low rectal temperature (<20°C) for nearly 10 h before gradually increasing to normal levels at approximately 18 h after the 5′-AMP injection. No obvious abnormalities were observed after recovery. 

### 4.2. Hypothermia Induced by 5′-AMP Decreases the Plasma Levels of Inflammatory Cytokines

**Figure 2:**
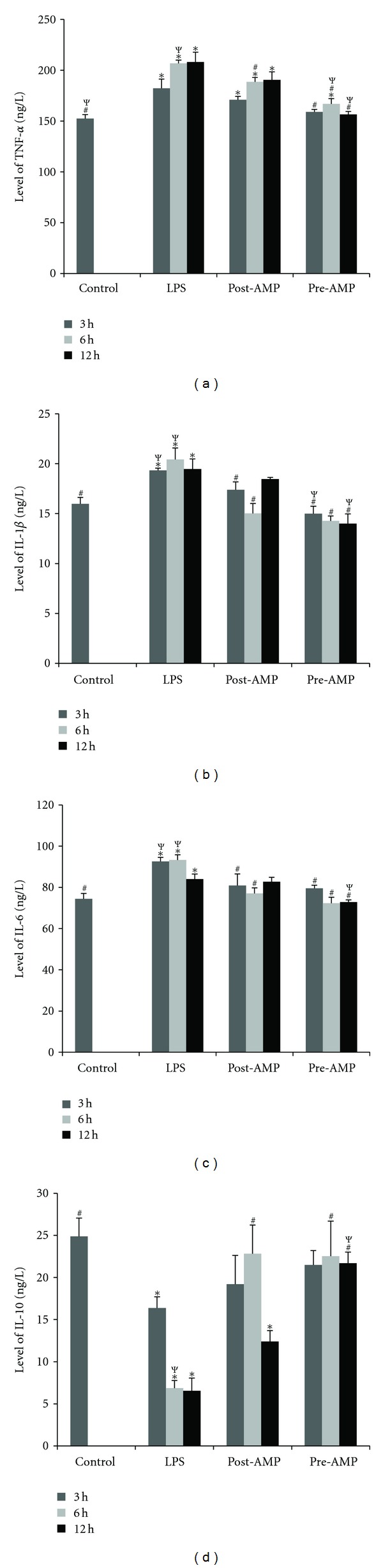
The plasma inflammatory factor levels.The plasma inflammatory factor levels in the rats were measured at 3 h, 6 h, and 12 h after LPS injection. (a) TNF-*α*; (b) IL-1*β*; (c) IL-6; (d) IL-10. Control group: no treatment; LPS group: endotoxemia rat model induced by LPS; Posttreatment group: endotoxemia rat model treated with 5′-AMP. Pretreatment group: rats pretreated with 5′-AMP prior to LPS treatment. *P < 0.05 versus control; ^#^ *P* < 0.05 versus LPS; ^Ψ^ *P* < 0.05 versus post-AMP.

Many experimental results have demonstrated that the levels of plasma cytokines such as TNF-*α*, IL-1*β*, and IL-6 are increased in LPS-induced inflammation [4, 13, 14]. Our results also showed that LPS could induce a significant increase of these cytokines when compared with the control group. As shown in Figure 2(a), we observed that hypothermia induced by 5′-AMP could significantly decrease the levels of TNF-*α* at 6 h after LPS injection in the post-AMP group (*P* < 0.05 versus LPS). In the pre-AMP group, hypothermia induced by 5′-AMP could also decrease the level of TNF-*α* at 3 h, 6 h, and 12 h after LPS injection (*P* < 0.05 versus LPS). We also observed that the rats in the pre-AMP group had lower levels of TNF-*α* when compared to the rats in the post-AMP group (*P* < 0.05 versus post-AMP). 

At 3 h and 6 h post-LPS injection, the rats in the post-AMP and pre-AMP groups had lower levels of IL-1*β* (*P* < 0.05 versus LPS) when compared with the rats in the LPS group. The rats in the pre-AMP group also had lower levels of IL-1*β* at 12 h after LPS injection. When compared with the post-AMP group, the 5′-AMP pretreatment could significantly decrease the levels of IL-1*β* (*P* < 0.05 versus post-AMP) at both 3 h and 12 h after LPS injection (Figure 2(b)).

Similar to the results seen for IL-1*β*, at 3 h and 6 h after LPS injection, the rats in the post-AMP and pre-AMP groups had lower levels of IL-6 (*P* < 0.05 versus LPS) when compared with the rats in the LPS group. The 5′-AMP pretreatment also led to a significant decrease in the levels of IL-6 at 12 h after LPS injection when compared with both the post-AMP or LPS groups (*P* < 0.05 versus LPS or post-AMP) (Figure 2(c)). 

As shown in Figure 2(d), we observed that the 5′-AMP pre-treatment and post-treatment could significantly increase the levels of IL-10 at 6 h post-LPS injection, when compared with the LPS group (*P* < 0.05 versus LPS). The 5′-AMP pre-treatment could also increase the level of IL-10 at 12 h post-LPS injection (*P* < 0.05 versus LPS). When compared with the pre-AMP group, the rats in the post-AMP group had lower levels of IL-10 at 12 h after LPS injection.

### 4.3. Hypothermia Induced by 5′-AMP Protects the Lungs against LPS-Induced Acute Injury

**Figure 3:**
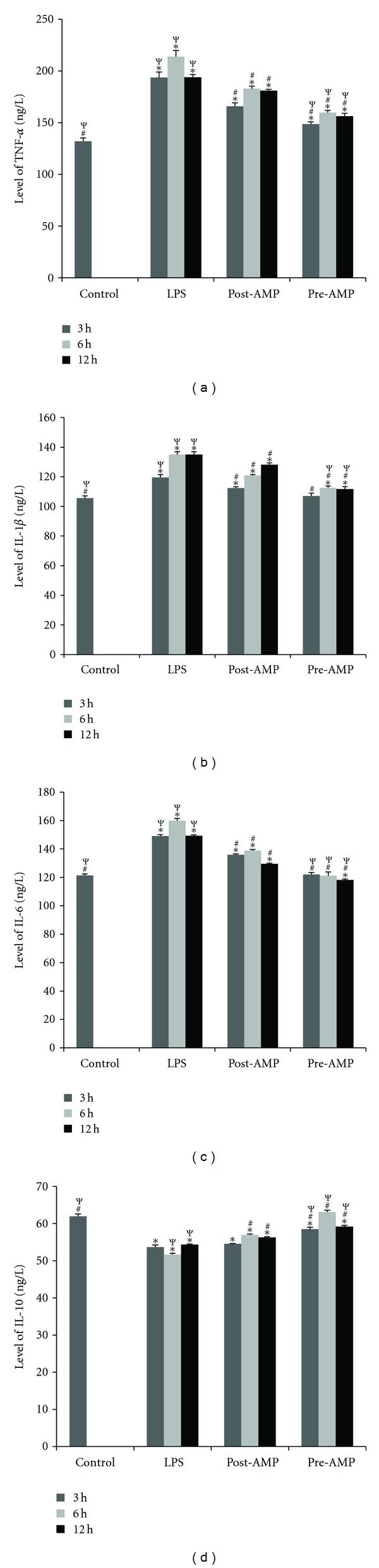
The BALF inflammatory factor levels. The BALF inflammatory factor levels in the rats were measured at 3 h, 6 h and 12 h after LPS injection. (a) TNF-*α*; (b) IL-1*β*; (c) IL-6; (d) IL-10. Control group: no treatment; LPS group: endotoxemia rat model induced by LPS; Posttreatment group: endotoxemia rat model treated with 5′-AMP. Pretreatment group: rats pretreated with 5′-AMP prior to LPS treatment. *P < 0.05 versus control; ^#^ *P* < 0.05 versus LPS; ^Ψ^ *P* < 0.05 versus post-AMP.

In the BALF samples, our results showed that LPS induced an observable increase of inflammatory cytokines, such as IL-1*β*, IL-6, and TNF-*α* and a significant decrease of anti-inflammation cytokines, such as IL-10. As shown in Figure 3(a), hypothermia induced by 5′-AMP could significantly lower the levels of TNF-*α* at 6 h after LPS injection in the post-AMP group (*P* < 0.05 versus LPS). In the pre-AMP group, hypothermia induced by 5′-AMP showed low levels of TNF-*α* at 3 h and 6 h after LPS injection and maintained these low levels even at 12 h after the injection (*P* < 0.05 versus LPS). The rats in the pre-AMP group had a significantly lower level of TNF-*α* when compared with the rats in the post-AMP group (*P* < 0.05 versus post-AMP). The levels of IL-1*β* and IL-6 (*P* < 0.05 versus LPS) were significantly reduced in the post-AMP and pre-AMP groups, and the rats in the pre-AMP group were able to maintain the concentrations at low levels (*P* < 0.05 versus post-AMP) (Figures 3(b) and 3(c)).

As shown in Figure 3(d), we observed that 5′-AMP pretreatment and posttreatment could increase the levels of IL-10 at 6 h and 12 h, when compared with the LPS group (*P* < 0.05 versus LPS). When compared with the pre-AMP group, rats in the post-AMP group had lower levels of IL-10 at 12 h after LPS injection.

**Figure 4:**
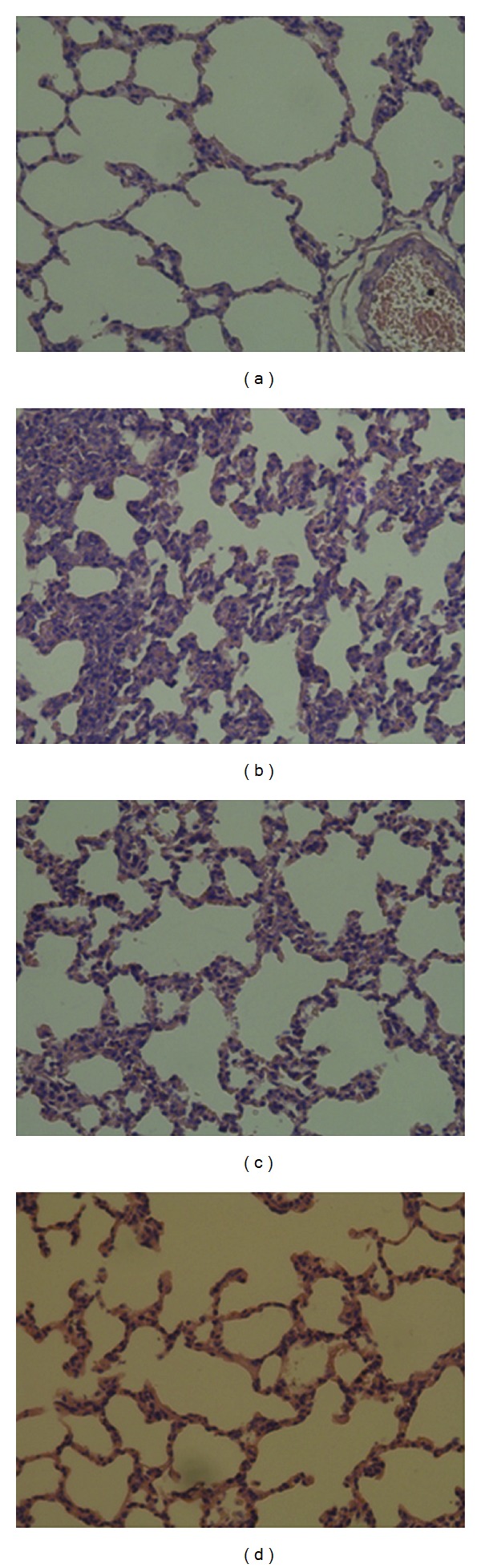
Photomicrographs of representative histological sections of the lung (400x magnification). (a) Control group: no treatment; (b) LPS group: endotoxemia rat model induced by LPS. (c) Posttreatment group: endotoxemia rat model treated with 5′-AMP. (d) Pretreatment group: rats pretreated with 5′-AMP prior to LPS treatment.

The histological results showed that AMP-induced hypothermia had the ability to protect the lung tissue from endotoxemic injury (Figure 4). When compared with the control group (Figure 4(a)), the lungs of the rats receiving LPS only (Figure 4(b)) had significant pathological changes, including: (1) broadening of the pulmonary interstitial tissue and (2) leukocyte infiltration, including monocytes and neutrophils. 

In the control group, we were unable to observe any pathological changes (Figure 4(a)), while in the LPS group (Figure 4(b)) we observed leukocyte infiltration and broadening of the pulmonary interstitial tissue. In the 5′-AMP posttreatment group (Figure 4(c)), we observed that endotoxemic rats treated with 5′-AMP exhibited only slight pathological changes to the lungs, such as a moderate broadening of the pulmonary interstitial tissue when compared with the control group. The rats in the 5′-AMP pretreatment group (Figure 4(d)) were very similar to the rats in the control group, with only minor pathological changes to the lungs.

## 5. Discussion

Many experimental studies and clinical experiences have shown that hypothermia can protect the brain from cerebral injury and downregulate inflammation [14–17]. In our study, we found that hypothermia induced by 5′-AMP has protective effects on endotoxemia, as demonstrated by the following criteria: (1) a significant reduction of IL-1*β* (*P* < 0.05), IL-6 (*P* < 0.05), TNF-*α* (*P* < 0.05) and an increase in IL-10 (*P* < 0.05) in the plasma and BALF levels in rats that were pre- and posttreated with 5′-AMP, when compared with rats injected with LPS only; (2) a significant inhibition of acute lung injury induced by LPS.

Many studies have shown that hypothermia can inhibit the expression of inflammatory cytokines and attenuate acute organ injury [18–20]. Our study also showed that the hypothermia induced by 5′-AMP could decrease the expression of inflammatory cytokines and attenuated acute organ injury. 5′-AMP is the circadian signal that mediates murine procolipase expression in the peripheral organs and induces torpor in mice [8]. Procolipase is a cofactor protein secreted by the pancreas. Colipase is the active form, and it is derived from procolipase through trypsin cleavage in the intestine [21]. It can regulate food intake in higher mammals, and the procolipase levels may be upregulated by the increase of 5′-AMP in the peripheral organs [22–24].

 Several clinical studies have indicated that acute lung injury (ALI) is provoked by an excess of proinflammatory cytokines, produced by the active neutrophils, that accumulate in the lung, which can directly damage the pulmonary capillary endothelial cells and induce the release of other inflammatory mediators [25]. In our study, pretreatment and posttreatment with 5′-AMP markedly reduced the lung inflammatory responses and improved the pulmonary histology. Proinflammatory cytokines, notably TNF-*α*, IL-1*β*, and IL-6, participate in the early development of inflammation and have been shown to play a crucial role in ALI. TNF-*α* and IL-1*β* are the primary cytokines responsible for initiating an acute inflammatory response [25]. We also found that the levels of TNF-*α*, IL-1*β*, and IL-6 in the BALF samples were dramatically increased after LPS induction. Pretreatment with 5′-AMP significantly lowered the production of the LPS-induced proinflammatory cytokines, and the cytokine levels were similar to those seen in the control group. Anti-inflammatory cytokines such as IL-10 are also produced during endotoxemia, which can down-regulate the production of proinflammatory cytokines and provide a key mechanism for limiting the inflammatory response in the lungs [25]. Based on our results, the inhibition of proinflammatory cytokines and the prevention of lung injury may be related to the increased BALF levels of IL-10, resulting from the pre-treatment of the rats with 5′-AMP 1 h prior to LPS administration. 

In conclusion, our findings provide direct evidence for the positive protection of 5′-AMP-induced hypothermia on inflammation in rats. We concluded that the hypothermia induced by 5′-AMP could inhibit inflammation and protect the lung against acute injury induced by LPS. The positive protection offered by pretreatment was more effective than that of posttreatment. Therefore, our studies provide both a theoretical basis and laboratory evidence for the supportive therapy of patients with septicemia though the optimal conditions and long-term effects of the hypothermia induced by 5′-AMP in this endotoxemia rat model still require further investigation.
